# Effects of narghile use on oral health: a comparative cross-sectional study

**DOI:** 10.1590/0103-644020266994

**Published:** 2026-10-19

**Authors:** Ana Carolina Elias Hanna, Ricardo Natã Fonseca Silva, Virgílio Moreira Roriz, Nádia do Lago Costa

**Affiliations:** 1 Department of Stomatologic Sciences, School of Dentistry, Federal University of Goias, Goiania, Goias, Brazil; 2 Department of Periodontology, School of Dentistry, Federal University of Goias, Goiania, Goias, Brazil

**Keywords:** Smoking water pipes, periodontitis, mouth mucosa, leukoplakia

## Abstract

The study aimed to assess the prevalence of oral mucosal lesions and periodontal disease in narghile users. A sample of narghile users (n=45) and non-users (n=45), matched by age and sex, was selected from the School of Dentistry at the Federal University of Goiás and tobacconists in Goiânia, Brazil. Demographic and socioeconomic data and characteristics of narghile users were collected through a structured questionnaire. The degree of dependence was assessed using the Fagerström Test for Nicotine Dependence. The median age was 25 (18 - 54) years in users and in non-users, with a predominance of males (80%, n=32). The median duration of use was 6.5 (0.5-33) years. Bleeding on probing was 29.72% (0.6-100) in users and 20.66% (0.2-63.4) in controls (p=0.799). Periodontitis was more frequent in users (n=19; 47.5%) than in controls (n=3; 7.5%) (p<0.001). An association was observed between the degree of dependence and periodontitis (p=0.002). In narghile users, 17 (42.5%) lesions were observed, including two leukoplakia cases. It was concluded that narghile users showed more oral cavity disorders compared to non-users, and an important association was found between the degree of dependence of narghile use and periodontitis

## Resumo

O objetivo deste estudo foi avaliar a prevalência de lesões da mucosa oral e doença periodontal em usuários de narguilé. Uma amostra de usuários de narguilé (n=45) e não usuários (n=45), pareados por idade e sexo, foi selecionada na Faculdade de Odontologia da Universidade Federal de Goiás e em tabacarias de Goiânia, Brasil. Dados demográficos, socioeconômicos e características dos usuários de narguilé foram coletados por meio de um questionário estruturado. O grau de dependência foi avaliado utilizando o Teste de Fagerström para Dependência de Nicotina. A mediana de idade foi de 25 (18-54) anos tanto em usuários quanto em não usuários. O tempo médio de uso foi de 6,5 (0,5-33) anos. O sangramento à sondagem teve uma mediana de 29,72% (0,6-100) em usuários e 20,66% (0,2-63,4) no grupo controle (p=0,799). A periodontite foi mais frequente em usuários (n=18; 41,4%) do que em controles (n=4; 9%) (p<0,001). Observou-se uma associação direta entre o grau de dependência e a periodontite (p=0,002). Nos usuários de narguilé, foram observadas 17 (42,5%) lesões orais, incluindo dois casos de leucoplasia, enquanto no grupo de não usuários foram encontradas 13 lesões. Concluiu-se que os usuários de narguilé apresentaram mais alterações na cavidade oral em comparação aos não usuários e que foi encontrada uma importante associação entre o grau de dependência do uso de narguilé e a periodontite. Além de ter sido observada uma ocorrência maior e estágios mais avançados de periodontite.

**Figure:**
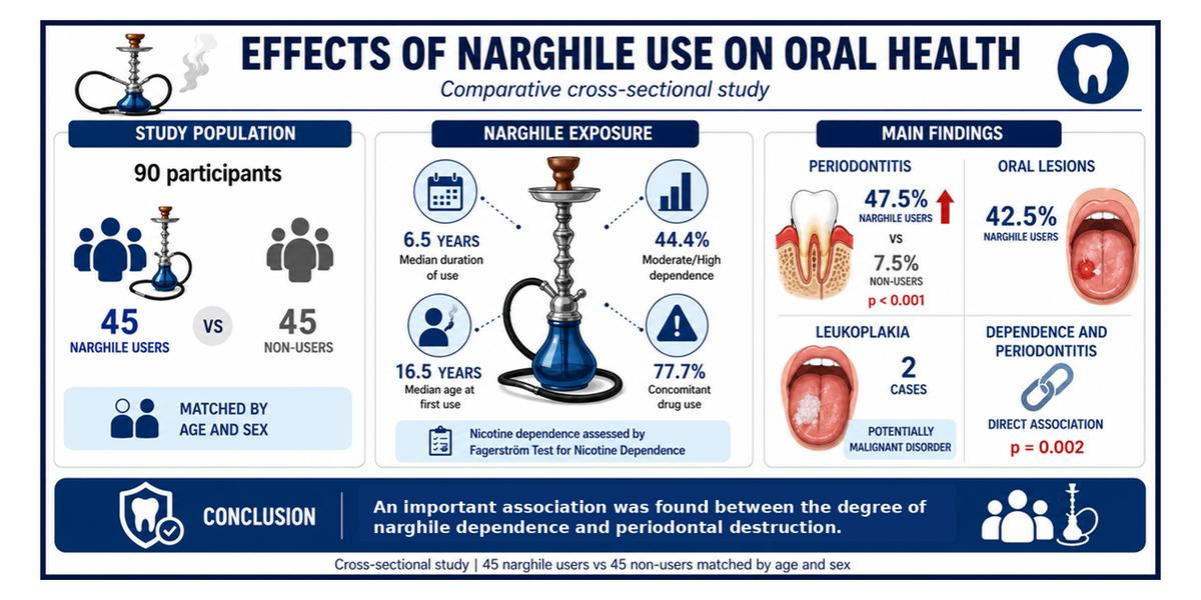

## Introduction

Although cigarette smoking is the predominant form of tobacco use almost all over the world, narghile use is responsible for a significant and growing share of smoking globally ^1^ ^,^ ^2^. In 2008, the Global Adult Tobacco Survey (GATS) reported a prevalence of use of 0.18% for men and 0.1% for women in Brazil ^1^. In 2015, the National School-Based Health Survey pointed to a significant increase in smoking prevalence, demonstrating that 6.1% of adolescents reported using other tobacco products, with 70% of this consumption being narghile ^3^ ^,^ ^4^. 

The narghile use has a socializing character that is elucidated and demonstrated as one of the main factors for its rapid popularization, especially among young people, who justify the entertainment with the mistaken discourse that it is a habit less harmful to health ^2^. Flavored smoke and contact with water provide the erroneous perception of lower toxic potential than cigarette smoke; however, several toxic substances, including human carcinogens, have already been identified in narghile products ^2^ ^,^ ^5^. Higher levels of carbon monoxide, nicotine, tar, pitch, and various flavorings were found in the smoke generated by narghile compared to conventional cigarettes ^6^ ^,^ ^7^.

Narghile users have reported a high degree of dependence and a higher incidence of chronic bronchitis, chronic obstructive pulmonary disease, hypertension, acute myocardial infarction, and stroke ^8^ ^,^ ^9^ ^,^ ^10^.

Few studies have been conducted to elucidate the effects of narghile use in the oral cavity. Still, the existing ones have shown a similar negative impact to those found in the use of conventional cigarettes ^11^ ^, (^ ^12^ ^),^ ^13^ ^),^ ^14^ ^12^ ^,^ ^15^. Narghile smoking has been associated with increased susceptibility to *Candida albicans* infections ^16^, oral cancer ^17^, and increased susceptibility to periodontal tissue destruction, generating periodontal disease and peri-implantitis ^18^ ^,^ ^19^ ^,^ ^20^, thereby causing increased probing depth, clinical attachment loss, and radiographic bone loss ^18^ ^,^ ^21^ ^,^ ^22^. 

Although data are sparse about the possible participation of the narghile in the oral mucosa and supporting tissues, the present study hypothesized that narghile users would have more periodontitis, potentially malignant lesions, and oral cancer than non-users. Therefore, the present study aimed to evaluate the prevalence of lesions in the oral mucosa and periodontal disease in narghile users.

## Materials and methods

### Study location, design and sample

This cross‐sectional study was conducted in the Center for Research in Oral Systemic Health, School of Dentistry, Federal University of Goiás, Goiânia, a city located in central Brazil. 

This study was designed in accordance with the STROBE guidelines for cross-sectional studies ^23^. A convenience sample of narghile users (n=45) and non-users (n=45), matched by age and sex during recruitment, was selected from the reception hall of the School of Dentistry at the Federal University of Goiás and from tobacconists. Participants were recruited between February 2019 and January 2023. The Research Ethics Committees at the Federal University of Goiás approved the study (CAAE: 83518118.9.0000.5083), ensuring anonymity and confidentiality of patient data. All participants or responsible relatives provided informed written consent to participate in the study. This study followed the principles of the Declaration of Helsinki.

Eligible participants were narghile users with regular use for more than six months, concomitant or not with the use of other forms of tobacco, with a minimum age of 18 years. For the control group (non-users), individuals who do not use any tobacco (neither narghile nor Marihuana) were included, matched by sex and age. Exclusion criteria for both groups included the inability to perform intraoral examinations and periodontal assessments, refusal to attend the study consultation for data collection, or inability to understand and respond to the forms used in the study. 

### Study variables and data collection instruments

### Independent Variables (Sociodemographic, economic data and narghile users characteristics)

An interviewer-administered questionnaire (ACEH and TMFL) was developed to collect information on demographic and socioeconomic characteristics for the main study, including age, sex, ethnicity, marital status, education level, family income, alcohol consumption, and general health status. The questionnaire also evaluated narghile use characteristics, including behavioral (frequency and use of other drugs), temporal (time and age of first use), and motivational variables (monthly expense, sharing of use, and reasons for experimenting). The degree of dependence was assessed using the Portuguese version of the Fagerström Test for Nicotine Dependence ^24^, previously adapted and validated in a Brazilian study for smokers. For the present study, this instrument was adapted for narghile use by replacing references to cigarette smoking with narghile use, while preserving the original structure, scoring system, and conceptual framework of the instrument. This approach maintained the instrument's original purpose: to assess the degree of tobacco dependence. Through a set of scored questions, this adapted version determines the interviewee's level of tobacco dependence.

### Dependent Variables: (oral lesions, periodontal findings)

The intraoral examination was performed by a specialist in oral medicine (NLC) qualified and trained for the systematic clinical analysis. The evaluation was performed with adequate lighting and a previously established sequence so that no anatomical structure of the oral cavity was left without inspection. All alterations found were registered according to the location and diagnosis hypothesis. When necessary, biopsy and histopathological examination were performed for treatment and final diagnosis.

The periodontal diagnosis was assessed using a 1 mm dental probe (University of North Carolina PCPUNC; Hu-Friedy, Chicago, IL, USA), with data recorded on a periodontal form. Periodontal measures evaluated were: probing depth (PD), clinical attachment level (CAL), and bleeding on probing. Patients were classified as healthy, with gingivitis, or with periodontitis, based on a classification of periodontal diseases and conditions ^25^ ^,^ ^26^. Gingival health was defined as < 10% bleeding sites with probing depth (PD) ≤ 3 mm. Gingivitis was defined as ≥ 10% bleeding sites with PD ≤ 3 mm, and periodontitis was defined as a detectable interdental clinical attachment level (CAL) at ≥ 2 non-adjacent teeth, or buccal or oral CAL ≥ 3 mm with periodontal pocket ≥ 3 mm at ≥ 2 teeth when not ascribed to non-periodontitis-related causes. Periodontitis was based on the severity and complexity of management, and the periodontitis cases were defined as stages of periodontitis (I, II, III and IV). Dental mobility was also evaluated ^25^.

Two periodontists (CAC and VMR) performed the periodontal assessment, helped by another assistant (RNFS). Inter- and intra-examiner reliability was assessed in a pilot analysis of PD and CAL measurements in 48-hour duplicate assessments of five volunteers with stage III generalized periodontitis, and acceptable agreement was achieved (intraclass correlation> 0.80).

### Data analysis

All statistical analyses were performed with IBM SPSS 22.0 (IBM Corporation, USA). Descriptive analyses were expressed as median values (minimum value‐maximum value). The Shapiro‐Wilk test indicates non‐normal distributions of numerical data, and the Mann‐Whitney test was used instead. Categorical variables were evaluated using the Pearson chi‐squared test. Results with p-values that were ≤ 0.05 were considered to be statistically significant.

## Results

During the 11-month recruitment and data collection period, 125 narghile users were invited to participate in the research. However, 72 were excluded due to refusal to participate, and eight participants were excluded due to the impossibility of performing oral examinations. Finally, 45 participants were assessed and included in the analysis. In the control group (nos-users), 45 participants were also selected according to the inclusion criteria.

The demographic and socioeconomic variables are displayed in Table 1. In the narghile users group, the age ranged from 18 to 54 years (median = 25). The majority of participants were male (82.2%), white (57.7%), single (86.6%), and had a university degree (48.8%), and most were from the lower socioeconomic stratum. The demographic and socioeconomic characteristics of the non-users group were similar to the narghile users group (p > 0.05). 

**Table 1:** Demographic data and clinical features of the narghile users group (n=45) and control group (n = 45).

Variables	Narghile users n(%)	Non-users n(%)	
Age (minimum ‐ maximum)	25 (18 - 54)	25 (18 - 54)	
Monthly family income^#^ (minimum ‐ maximum)	USD 800.00; (320.00 - 8.000)	USD 800.00; (80.00 - 6.000)	p > 0.05*
Gender			p > 0.05^†^
Female	8 (17.7)	8 (17.7)	
Male	37 (82.2)	37 (82.2)	
Race			p > 0.05^†^
White	26 (57.7)	29 (64.4)	
Brown	13 (28.8)	11 (24.4)	
Black	6 (12.5)	5(11.1)	
Educational level			p > 0.05^†^
Elementary and middle school	17 (37.7)	3 (6.6)	
Incomplete higher education	22 (48.8)	28 (62.2)	
High school	5 (11.1)	9 (20)	
Higher education	1 (22)	5 (11.1)	
Nationality			p > 0.05^†^
Brazilian	43 (95.4)	45(100)	
Foreigner	2 (94.4)	0 (0)	
Marital status			p > 0.05^†^
Single	39 (86.6)	43 (95.5)	
Married	4 (8.8)	2 (4.4)	
Divorced	2 (4.4)	0 (0)	
Habitation			p > 0.05^†^
Parents	12 (26.6)	9 (20)	
Famíly	27(60)	20 (44.4)	
Single	5 (11.1)	13 (28.8)	
Others	1(2.2)	3 (6.6)	

# Data collected in Brazilian reais and converted to US dollars using an exchange rate of R$5 to USD1.

*Mann-Whitney test

† Pearson chi‐squared test

Regarding narghile use characteristics, the habit's median duration was 6.5 (0.5 - 33) years, with a median age of initial experimentation of 16.5 (12 - 33) years. Houses and tobacconists were the most frequent places of shisha use (57.7%; n=26), and 97.7% (n=44) of users reported sharing it with others. About frequency of use, 22 users reported using hookah 3-5 times a week (48.8%), and 35 (77.7%) used it concomitantly with different drugs. Monthly spending on the habit ranged from USD 10.0 to USD 240.0 with a median of USD 50.3. The degree of dependence, evaluated by the adapted Fagerström test, revealed that 44.4% (n=20) of users had degree III or higher dependence. All the characteristics of narghile use are shown in Table 2.

Assessment of periodontal status revealed that the median bleeding on probing was similar between narghile users (29.72) and the control group (20.66) (p = 0.799, Pearson chi‐squared test). Tooth mobility was observed in both groups: in five participants of narghile users and in one participant of non-users. There was a higher occurrence of periodontitis in narghile users (40%; n=18) compared to the control group (8.8%; n=4) (p<0.001), and only in the group of users was periodontitis stage III found ^4^. The bivariate comparison revealed a direct association between the degree of dependence and the occurrence of periodontitis, where eight patients diagnosed with periodontitis also had degree III or higher dependence (p=0.002, Pearson chi‐squared test). Details on periodontal conditions are described in Table 3.

**Table 2:** Features of using narghile.

Variables	n (%)
Frequency of use	
1-2 times per week	3 (6,7)
3-5 times per week	24 (53,3)
> 5 times per week	18 (40)
Shared use	
Yes	44 (97.7)
No	1 (2.2)
Reasons for experimenting	45 (100)
Curiosity	18 (40)
Influence of friends	14 (31.1)
Family culture	5 (11.1)
Others	8 (17.7)
Association with other drugs	35 (100)
Cigarette	7 (20)
Straw cigarette	5 (14.2)
Organic Tobacco	5 (14.2)
Viper	6 (17.1)
Marihuana	12 (34.2)
Alcohol	19 (55)
Degree of dependence according to the Fagerström Test	
Score I (very low dependence)	19 (42,2)
Score II (low dependence )	18 (40,2)
Score III (moderate dependence )	4(8,8)
Score IV (high dependence )	2 (4,4)
Score V (very high dependence )	2 (4,4)
Places of use	
Home	5 (11,1)
Work	2 (4,4)
Tobacco shop	12 (26,6)
Home and Tobacco shop	26 (57,7)
Time of use (median)	6.5 years ;
(minimum-maximum)	(0.5 - 33)
Monthly expense with use^#^ (median)	USD 50.30;
(minimum ‐ maximum)	(10.00 - 240.00)
Age of first use (median)	16.5 years
(minimum-maximum)	(12 - 33);

# Data collected in Brazilian reais and converted to US dollars using an exchange rate of R$5 to USD1.

**Table 3:** Periodontal status of narghile users (n=45) and control group (n=45).

Variables	Narghile Users n(%)	Non-users n(%)	p value
Bleeding on probing (minimum ‐ maximum)	29.72 (0.6 - 100)	20.66; (0.2 - 63.4)	0.799*
Periodontal Diagnosis			
Periodontal health	1 (2.2)	7 (15.9)	0.002^†^
Gingivitis	25 (56.8)	34 (77.2)	
Periodontitis stage I	8 (18.8)	3 (6.8)	
Periodontitis stage II	6 (13.6)	1 (2.2)	
Periodontitis stage III	4 (9.0)	0 (0)	
Prognosis			
Favorable	43 (97.7)	45 (100)	
Unfavorable	1 (2.2)	0 (0)	

*Mann-Whitney test

† Pearson chi‐squared test

Seventeen (42.5%) lesions were observed in narghile users in the intraoral examination. Of these, two were diagnosed as leukoplakia and the remaining 15 as lesions of traumatic or reactional origin. In the control group, 13 (32,5%) reactional lesions were identified. The main site of involvement was the buccal mucosa of the cheek in both groups.

## Discussion

This clinical study reported the association between chronic narghile use and oral health conditions. Our findings suggest that poor periodontal status is associated with the habit of smoking narghile, with a high degree of dependence considered a significant factor associated with periodontitis as a whole ^26^. Furthermore, narghile users can also manifest potentially malignant disorders, such as leukoplakia.

The worrying scenario of the prevalence and frequency of narghile use in Brazil was demonstrated in an important study ^27^, and in the present study it could be observed that 55% of the participants reported using it more than five times a week and 32.5% with degrees of dependence above III. In addition, the sociodemographic and economic characteristics found in the present study corroborate previous findings, with the vast majority of users being young, male, and with high family income ^27^.

Regarding the effect of narghile use on periodontal tissues, the literature shows a scarcity of studies; however, the studies already carried out indicate that the habit of smoking, regardless of the product, is related to periodontal attachment loss ^28^. Ramôa et al. (2017) found higher rates of attachment loss, probing depth, plaque index, and alveolar bone crest loss in narghile users than in non-smokers ^12^. Bergström (2004) demonstrated that narghile smokers with a high degree of oral hygiene had three times greater bone and tooth loss than non-smokers during a 10-year study period ^29^. The results of periodontal indices of narghile smokers, according to Javed et al. (2016), were similar to those found in conventional cigarette smokers, in which several studies have already elucidated the periodontal consequences ^21^.

However, an important and distinct finding in the present study was that there was no significant decrease in bleeding rate in narghile users. Described in several studies on the influence of cigarettes on periodontal tissues, the vasoconstrictor effect of nicotine, a factor often confounding the correct periodontal diagnosis, was expected to be found in narghile users since most of the effects are similar ^30^. In the present study, on the contrary, the inflammatory response to the accumulation of plaque in the narghile user was as severe as that found in non-users. However, periodontal destruction was significantly greater than in the control group.

In the present study, a higher loss of attachment was found in narghile smokers, associated with a higher prevalence of periodontitis, to varying degrees, compared to never smokers. It was noticed that there was more important periodontal destruction in the narghile users. These results corroborate a previous study, which found a higher prevalence of periodontal disease among narghile users and cigarette smokers than non-smokers ^31^.

Among the various factors associated with the worsening of periodontal disease and systemic changes, smoking and other forms of tobacco use stand out due to their substantial impact on the loss of periodontal tissues. ^28^ ^,^ ^29^ ^,^ ^30^ ^,^ ^31^. More studies with a larger sample size are recommended to compare the strong trends of the relationship between periodontal disease, malignant and potentially malignant lesions, and narghile smoking.

Carcinogenic substances similar to those found in conventional cigarettes have also been identified in narghile smoke, including carbon monoxide (CO), volatile polycyclic aldehydes, and hydrocarbons ^6^ ^,^ ^32^. In conventional cigarette smokers, studies have demonstrated the manifestations of potentially malignant (leukoplakia) and malignant (oral squamous cell carcinoma - OSCC) oral lesions. Due to the cigarette-like carcinogenic and toxic composition and even higher concentrations of some substances in a single session, there is a possibility that narghile smoking may be associated with the occurrence of leukoplakia and oral squamous cell carcinoma ^33^ ^,^ ^34^. In the present study, two cases of leukoplakia were found in two narghile users. This could be a warning sign, presenting an important opportunity to explore this topic in future studies that seek to prospectively follow these users over the long term, looking for evidence demonstrating the significant association between narghile use and the occurrence of other manifestations of potentially malignant oral lesions or even oral cancer.

As a limitation of the present study, concomitant use of narghile with other substances was observed, which may act as a potential confounder. In our sample, 35 participants reported concomitant use, mainly with marijuana, cigarettes, and organic tobacco, in addition to alcohol consumption reported by them. Notably, the use of narghile has been consistently associated with the use of other tobacco products and psychoactive substances, particularly among adolescents and young adults, including cigarettes, alcohol, and cannabis, often within patterns of polytabacco and polysubstance use ^35^ ^,^ ^36^. Another important limitation is that the Fagerström Test for Nicotine Dependence, although previously adapted and validated for Brazilian smokers, was adapted in this study for narghile use by replacing references to cigarette smoking with narghile use, since there is no validated instrument specifically designed for narghile users. Therefore, the lack of prior validation of this adapted version for narghile users should be considered when interpreting the findings. 

However, as this is an observational study and due to the worldwide trend of using more than one drug among users, especially young people, future studies that systematically assess the combined impact of multiple substance use on oral health should be encouraged.

## Conclusion

Within the limitations of this clinical study, it can be concluded that narghile users showed more oral cavity disorders compared to non-users. Furthermore, an important association was found between the degree of dependence on narghile use and periodontal destruction, demonstrating a much higher and more advanced occurrence of periodontitis in these users. In this way, like other forms of tobacco use, narghile users deserve special attention in the oral assessment guidelines on oral health and public health policies.

## Data Availability

The research data are available within the article.
